# Quackery

**Published:** 1851-01

**Authors:** 


*Quackery.*—We propose, from time to time, to speak of the different forms of Quackery, in the profession as well as out of it. In the Boston Transcript we find some articles upon this subject, from which we offer extracts instead of our own thoughts. It is understood that the writer is a lawyer, his *nomme de plume* being "a sexton of the old school." In No. 113, of "dealing with the dead," he says:

"There is nothing marvellous in the existence of quackery, if we recognize the maxim of M. Sorbiere, in his *Relation d'une Voyage en Angleterre*, p. 155, *homo est animal credulum et mendax*—man is a credulous and lying animal. David said, that all men were liars; but, as this is found in one of his lyrics, and he admits that he uttered it in haste, it may be fairly carried to the account of *poetica licentia.* With no more, however, than a moderate allowance for man's notorious diathesis, towards lying for pleasnre or profit, it is truly wonderful that credulity should preserve its relative level, as it does, and ever has done, since the world began. Many, who will not go an inch with the Almighty, without a sign, will deliver their noses, for safe keeping, into the hands of a charlatan, and be lead by him, blind-fold to the charnel house. Take away credulity, and the world would speedily prove an exhausted receiver for all manner of quackery."

The following, we apprehend, gives the true explanation of the fact that educated men are so often found to encourage empiricism. We once availed ourselves of the columns of a religious newspaper to protest against a laudation of hydropathy which had appeared in the same journal. Afterwards we received a dogmatical *admonetur* from the reverend editor, which not only afforded us amusement, but convinced us that he too knew nothing about the matter.

Ignorance is the hot-bed of credulity. This axiom is not the less respectable because the greatest philosophers occasionally place confidence in the veriest fools, and do their bidding, Wise and learned men, beyond the pale of their professional pursuits, or peculiar studies, are, very frequently, the simplest of simple folks—*non omnia possumus omnes.* Ignorance must be very common, for a vast majority of the human race have not proceeded so far in the great volume of wisdom and knowledge, as that profitable but humiliating chapter, whose perusal is likely to stimulate their energies by convincing them that they are of yesterday and know nothing, Credulity must, therefore, be very common.

But our sexton continues to discourse in this way:

In a dissertation before the Medical Society, in June, 1828, Dr. George Cheyne Shattuck, after setting forth a melancholy catalogue of the trouble and perplexities of the medical profession, concludes by saying, that, "all these trials to which the physician is subjected, do not equal that which proceeds from the *uncertainty* of the healing art." When we contrast this candid avowal from an accomplished and experienced physician, with the splendid promises and infallible assurances of empirics—with their balms of Gilead, panaceas and elixirs of everlasting life—we cannot marvel that the larger part of all the invalids, in this uncertain and credulous world, fly from those conservative professors who promise nothing, to such as will assure them of a perfect relief from their maladies, no matter how complicated or chronic they may be—with four words of inspiriring import—NO CURE, NO PAY.

I am no physician; my opinion, therefore, is not presented *ex cathedra:* but the averment of Dr. Shattuck is, I presume, to be viewed in no other light, than as the opinion of an honorable man, who would rather claim too little than too much for his own profession; who would rather perform more than he has promised, than promise more than he can perform. If the regularly bred and educated physician complains of uncertainty, none but a madman would seek for its opposite in the palace or the kennel of a quack —for the charlatan may occasionally be found in either.

The first thing to be done, I suppose, by the regular doctor, is to ascertain what the disease is. This, I believe, is the very last thing thought of by the charlatan. He is spared the labor of all pathological inquiry, for all his medicines are fortunately, panaceas. Thus he administers a medicine for the gout; the patient does not happen to have the gout, but the gravel; it is the same thing; for the physic, like our almanacs, was calculated for different meridians."

It is most refreshing to us, to find educated men thus speaking out the truth on matters concerning which they often err. We would desire to express our thanks for every blow struck in our favor by those who ought always to be with us, but are too ready to oppose. From the nature of the case, their influence is far greater than that of a physician, to whom, perhaps, not without seeming justice, it is objected that it is his craft that is in danger.—*New Hampshire Med. Jour.*

